# Compatibility of *Beauveria bassiana* and a Plant Secondary Metabolite: A Novel Modeling Approach to Invade Host Defense for Effective Control of *Oligonychus afrasiaticus* (McGregor) on Date Palms

**DOI:** 10.3390/jof7050334

**Published:** 2021-04-26

**Authors:** Abid Hussain

**Affiliations:** 1 Institute of Research and Consultancy, King Faisal University, Hofuf, Al-Ahsa 31982, Saudi Arabia; abhussain@kfu.edu.sa or solvia_aah@yahoo.com; Tel.: +966-566989571; 2 Ministry of Environment, Water and Agriculture, Riyadh 11442, Saudi Arabia

**Keywords:** antagonism, antioxidants, biological control, biological index, date palm dust mites, entomopathogenic fungi, joint toxicity, synergism, toxin-pathogen interaction, (+)-α-pinene

## Abstract

*Oligonychus afrasiaticus* (McGregor) is an important pest causing substantial economic losses to date palm fruits (dates). The application of mycopathogens with plant secondary metabolites, which may proceed synergistically is thus essential to augment sustainable management strategy for *O. afrasiaticus*. In this regard, extensive laboratory experimentation involving compatibility, synergism, and host defense was performed to develop stable pest management option. The toxin-pathogen compatibility assay results revealed compatible interaction (biological index = 79–95) of *B. bassiana* ARSEF 8465 against each tested concentration of commercially available (+)-α-Pinene that provide the opportunity to further explore the time and concentration dependent mortality and defense related enzymatic regulation analysis. The time-mortality response assays that mainly comprised of various proportions of *B. bassiana* ARSEF 8465 and (+)-α-Pinene revealed that the sole application of *B. bassiana* ARSEF 8465 (LC_50_ = 19.16 mg/mL), and (+)-α-Pinene (3.41 mg/mL) found to be least lethal compared with joint applications (LC_50_ ranged from 1.32–7.06 mg/mL). The treatments complied under Scheme IV (80% (+)-α-Pinene: 20% *B. bassiana* ARSEF 8465 Conidia) led to strong synergistic interaction (joint toxicity = 755). In addition, synergistic interactions greatly induced enzymatic activities of the studied antioxidants (CAT and SOD), and defense-related enzymes (GST and AchE). We concluded that join application of *B. bassiana* ARSEF 8465 and (+)-α-Pinene is a promising option for controlling *Oligonychus afrasiaticus* populations.

## 1. Introduction

The date palm (*Phoenix dactylifera* L.) is an economically important palm species grown in the arid, sub-tropical, and tropical regions. It has the tendency to tolerate not only the harsh extremely high desert temperature but also resistant to drought and salinity. Globally, Saudi Arabia stands second for dates production after Egypt. Their production in Saudi Arabia according to United Nations Food and Agriculture statistics reached 1.54 million tons/year during 2019 that is 16.97% of the total world’s dates production [1]. However, Saudi Arabia being the major producer of dates is conscious about the loss of date palm fruits due to the infestations of various pests. The reduced yield that mainly leads to low production is mainly because of degradation of date palms due to trunk pests [2,3,4], and the associated date palm fruit pests’ infestations [5,6]. The *Oligonychus afrasiaticus* (Acari: Tetranychidae), is an economically important pest of the fruit of date palms in Saudi Arabia [6], along with some other countries including Algeria, Egypt, Iran, Iraq, Israel, Libya, Mauritania, Oman, Tunisia, and Yemen [7,8,9,10,11,12,13,14,15,16]. The environmental conditions prevailing in these areas greatly favored the growth and development of *O. afrasiaticus* on date palm fruits [17]. At the advent of fruit formation, *O. afrasiaticus* infestations start on the date palms by spinning the web around the bunches of dates [18]. These webs collect the dust along with the exuviae from their different developmental stages resulting dusty appearance of the infested bunches of dates. As a consequence, they start egg laying with their full potential and their population multiply logarithmically. These webs remain intact till later stages of the development of dates resulting decline in growth and development of date palm fruit. Such damages have caused subsequent crop losses and rendering their fruits unfit for human consumption [2,5,19].

The control of *O. afrasiaticus* involved the application of synthetic acaricides. However, the residues associated with acaricides are harmful for the consumers and also responsible for environmental pollution. Furthermore, the indiscriminate use of synthetic acaricides is restricted due to the deleterious effects on non-target animals. These shortcomings have led to find other efficient, safe, and environmentally friendlier alternatives to control *O. afrasiaticus* populations. An important component of biological control agents is the use of entomopathogenic fungi. These naturally occurring entomopathogenic fungi are considered important substitutes for acaricides. Most of the entomopathogenic fungi belong to Entomophthorales and Hypocreales. According to an estimate, around 700 taxa of fungi with more than 13,000 isolates of entomopathogenic fungal species isolated from 1300 different hosts have been reported to infect pests including aphids [20], beetles [21,22], cockroaches [23], grass-hoppers [24], mosquitoes, lepidopterous [25,26], termites [27], thrips [28], weevils [29], whitefly [30]. However, the slow mode of action of fungal isolates along with reduced virulence over the period of time is found to be the main hurdle in the way of their commercialization. In this regard, the concept of incorporation of plant secondary metabolites into the conidial suspension is gaining momentum in the field of eco-friendly pest management. In this regard, a polyphenolic terpene hydrocarbon (+)-α-Pinene is very much pertinent to the current study due to known toxicity against various pests including head lice [31], *Sitophilus zeamais* Motschulsky [32], *Spodoptera litura* and *Achaea janata* [33]. In the past, numerous studies have been conducted on the compatibility and joint action of conidial suspensions with plant-based active natural products [34,35,36], and their findings revealed enhanced joint treatment response. The addition of plant secondary metabolites for developing biorational pest management technologies is gaining interest nowadays because of numerous modes of action such as cuticle disruption, molting inhibition, respiratory failure, growth, and fecundity reduction. In addition, joint application of plant secondary metabolites with conidial suspensions is advantageous over synthetic pesticides because they are environmentally friendly and delay the development of resistance among the target pest populations.

In the past, little information is available on the joint action of plant secondary metabolites with conidial suspension against *O. afrasiaticus* [6,37]. These preliminary studies showed the tremendous potential of plant secondary compounds and emphasized on the exploration of more isolates and plant secondary metabolites to develop the compatible formulation for the eco-friendly control of *O. afrasiaticus*. The current study is mainly aimed at (1) evaluating the compatibility of conidial suspensions with (+)-α-Pinene by compatibility assays; (2) testing the acaricidal potential of a selected plant secondary metabolite (+)-α-Pinene, and *B. bassiana* ARSEF 8465 by concentration mortality response bioassays; (3) calculating the joint toxicity index to sort out the nature of their interaction; (4) exploring the treatment effects on the regulation of defense-related enzymes controlling physiological mechanisms of date palm dust mites, in order to develop a synergistic interaction with improved efficacy for the effective management of *O. afrasiaticus* populations infesting date palm fruits.

## 2. Materials and Methods

### 2.1. Microbial Culture

The culture of *Beauveria bassiana* ARSEF 8465 originally isolated from *Doru lineare* during December 2006 from Argentina was procured from the ARSEF collections of USDA-ARS. The *Beauveria bassiana* ARSEF 8465 isolate selection for this particular study was mainly on the basis of preliminary laboratory bioassays that showed virulence against *O. afrasiaticus*. The cultures of *B. bassiana* ARSEF 8465 isolate were maintained on Petri plates provided with potato dextrose agar in complete darkness for 24 d at 25 ± 0.5 °C. For bioassays, conidial suspensions were prepared in Tween 80 at a strength of 0.05% by Neubauer hemocytometer under a compound microscope.

### 2.2. Oligonychus afrasiaticus

The field collected date palm dust mite populations from National Center for Palms and Dates, Al-Ahsa, Saudi Arabia were maintained until experimentation in a photoperiod of 16 h light and 8 h darkness (62.5 ± 12.5% RH; 25 ± 1 °C). Overall, five different populations of date palm dust mites were directly collected from the mentioned locality to prepare different replicates for this study.

### 2.3. (+)-α-Pinene

**Figure 1:**
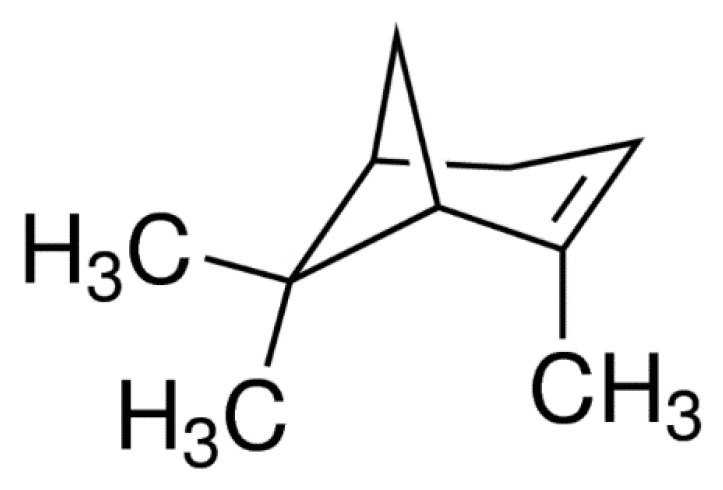
Chemical structure of (+)-α-Pinene (CAS Number 7785-70-8).

The compound (+)-α-Pinene as plant secondary metabolite was purchased from Sigma Aldrich London, UK (Cat # 268070) for toxicity, compatibility, toxin-pathogen interactions, and host physiological defense related enzymatic analysis. The stock solution of (+)-α-Pinene (Figure 1) was prepared in ethyl alcohol.

### 2.4. Fungus-Toxin Compatibility Assays

The compatibility of plant secondary metabolite (+)-α-Pinene with the conidial suspension (1 × 10^7^ conidia/mL) of *B. bassiana* ARSEF 8465 was evaluated by calculating the biological index (BI) through below mentioned formula [38]. The important fungal parameters such as vegetative growth; conidial germination; and conidiation of the studied fungal culture were determined accordingly to calculate biological index. The nature of interaction was determined by following the criterion mentioned in Table 1.

Biological Index = [47 × VG + 43 × SP + 10 × GR]/100

#### 2.4.1. Germination (GR)

In the first part of compatibility assays, impact of various concentrations (0.7, 1.4, 2.1, 2.8, and 3.5 mg/mL) of (+)-α-Pinene on the *B. bassiana* ARSEF 8465 germination percentage was evaluated. In brief, sterilized PDA after cooling at 50 °C was used to prepare above mentioned five different concentrations of (+)-α-Pinene, separately. Ten Petri dishes (*n* = 10 replicates) each by pouring 10 mL of potato dextrose agar supplemented with each tested concentration of (+)-α-Pinene was prepared under a laminar airflow cabinet. The control treatment petri dishes were prepared using PDA with 1% ethyl alcohol. After cooling the petri dishes, 200 µL conidial suspension of *B. bassiana* ARSEF 8465 (1 × 10^7^ conidia/mL) was used to inoculate all experimental units. After sealing with parafilm, all the inoculated petri dishes were incubated (25 ± 0.5 °C) in complete darkness. After 12 h post-inoculation, percent conidial germination was recorded under compound microscope after counting 100 conidia from ten different fields of vision. Germination data were adjusted through arcsine transformation prior to one way analysis of variance (ANOVA) to analyze percent conidial germination (GR), and significant differences among them by applying Fisher’s LSD test [39].

#### 2.4.2. Vegetative Growth (VG)

In the second part of compatibility assays, effect of (+)-α-Pinene at different concentrations was evaluated against the vegetative growth of *B. bassiana* ARSEF 8465 as described in our previous study [6]. Five different above mentioned concentrations of (+)-α-Pinene were prepared in PDA. For each concentration, ten petri dishes (*n* = 10 replicates) were poured with PDA supplemented with each concentration of (+)-α-Pinene. The control treatment was prepared by using PDA with 1% ethyl alcohol. Each experimental unit petri plate was inoculated by micropipette with five microliter conidial suspension of *B. bassiana* ARSEF 8465 (1 × 10^7^ conidia/mL). Two weeks post-inoculation at 25 ± 0.5 °C, vegetative growth (VG) data of the fungal growth were measured with the help of a transparent ruler to calculate their perpendicular radial lengths. One way Analysis of variance (ANOVA) was used to analyze vegetative growth of *B. bassiana* ARSEF 8465, and significant differences by Fisher’s LSD test [39].

#### 2.4.3. Conidiation (SP)

In the third part of compatibility assays, conidiation of *B. bassiana* ARSEF 8465 exposed with above mentioned concentrations of (+)-α-Pinene was determined from 15 mm colony suspended in Tween 80 at a strength of 0.05%. The conidiation was determined from above mentioned two weeks old fungal cultures used to record vegetative growth. The conidial count of each experimental unit suspension was made by a Neubauer hemocytometer under a compound microscope. The conidiation data were analyzed by one way ANOVA, and significant differences by Fisher’s LSD test [39].

### 2.5. Fungus-Toxin Synergism Bioassays

The impacts of the sole application of (+)-α-Pinene and *B. bassiana* ARSEF 8465, and their different combinations indicated in Table 2 were evaluated against *O. afrasiaticus* deutronymphs stage [37]. Different combination schemes I-IV (Table 2) were prepared by jointly mixing them in different proportions in order to precisely attain maximum treatment effect. In brief, each treatment solution with a particular proportion indicated in Table 2 was prepared using the respective solvent i.e., conidia in 0.05% Tween 80, while (+)-α-Pinene in 1% ethyl alcohol. Acaricide free date palm leaf-disks (75 mm L × 40 mm W), after washing with sterile distilled water were air-dried for subsequent dipping with forceps in treatment solutions, separately. The treated leaf-disks after drying were placed in a petri dish provided with sterile water-soaked cotton wool. In each petri dish, three leaves were placed, and each petri dish was considered as a single replicate. The edges of each treated leaf-disk were surrounded with water-saturated cotton strips in order to avoid the escape of mites. Fifty *O. afrasiaticus* were transferred by camel hair brush separately on each treated leaf-disk. Five replicates per treatment were prepared likewise by incubating each experimental unit at 62.5 ± 12.5% RH; 25 ± 1 °C. Similarly, control treatments were also prepared likewise using their respective solvents. Mortality data for each experimental unit was recorded after every 24 h under the microscope. Abbott formulae was used to correct the percent mortality data of *O. afrasiaticus* [40]. The corrected cumulative mortality data were angularly transformed for LC_50_ determination by Probit analysis [41], for subsequent determination of the nature of interaction through Joint Toxicity mentioned by AlJabr et al. [37]. However, significant differences were compared by LSD test after analyzing transformed cumulative mortality data by repeated measures ANOVA [39].

### 2.6. Response of Fungus-Toxin Interactions on the Target Host Defense-Related Enzymatic Regulation

The date palm leaf-disks as mentioned in Section 2.5 were prepared by dipping them into their respective solution indicated in Table 2 for exploring the defense mechanism of *O. afrasiaticus*. The exposed individuals of the five replicates including control treatments were incubated until 96 h post-exposure on controlled conditions. Approximately 500 alive date palm dust mites after 96 h were removed to prepare their solution in the pH 7.0 potassium phosphate buffer (0.05M). After centrifugation, each treatment supernatant was used to quantify protein at 595 nm absorbance [42]. The enzymatic analysis were performed by following the methodologies for catalase (CAT), superoxide dismutase (SODs), acetylcholinesterase (AChE), and glutathione S-transferase (GST) provided in detail in our previous study [43]. Each treatment sample supernatant that served as enzymatic source of each treatment was used to calculate AChE activities by following methodology provided by the supplier Abcam (Cat #. ab13887; Shanghai, China). The GST activity was calculated following the standard methodology of Habig et al. [44], and recording the enzymatic response by VersaMax Microplate Reader at 340 nm. The enzymatic activities of antioxidants including SODs (Cat # 19160-1KT-F), and CAT (Cat # CAT100-1KT) were calculated by following the standard methodologies provided by the Sigma Aldrich, London, UK. The percentage data about enzymatic activities generated relative to control treatment were analyzed by two-factor factorial analysis. Significant differences among means were compared by LSD Fisher’s test [39].

## 3. Results

### 3.1. Pathogen-Toxin Compatibility Response Analysis

All the tested concentrations of (+)-α-Pinene affected the studied parameters of *B. bassiana* ARSEF 8465, and revealed directly proportion concentration-dependent response (Table 3). There were significant differences (*F* = 3.43; *p* = 0.009) in percent germination of *B. bassiana* ARSEF 8465 conidia recorded 12 h post-inoculation in the presence of all tested concentrations of (+)-α-Pinene. Overall, we have calculated negligible germination inhibition response that ranged from 0.91% for the lowest concentration (0.7 mg/mL), to 3.74% for the highest concentration of (+)-α-Pinene (3.5 mg/mL), compared to the control treatment.

Similarly, vegetative growth also revealed similar inhibition patterns of *B. bassiana* ARSEF 8465 in response to different concentrations of (+)-α-Pinene such as 0.7, 1.4, 2.1, 2.8, 3.5 mg/mL resulting 1.36%, 4.04%, 7.16%, 11.32%, and 14.32% inhibition compared to control treatment, respectively. However, mean vegetative growth of *B. bassiana* ARSEF 8465 recorded against each tested concentration showed highly significant differences (*F* = 6.33; *p* = 0.0001) as shown in Table 3. 

Conidiation of *B. bassiana* ARSEF 8465 also showed similar pattern in response to different concentrations of (+)-α-Pinene. Results exhibited significant differences among treatments (*F* = 3.12; *p* = 0.0001). Furthermore, we have recorded 5.26%, 11.84%, 15.79%, 23.68%, 30.26% conidiation inhibited against various concentrations of (+)-α-Pinene such as 0.7, 1.4, 2.1, 2.8, and 3.5 mg/mL, respectively.

According to the biological index calculated from various biological parameters exhibited that *B. bassiana* ARSEF 8465 response at all tested concentrations of (+)-α-Pinene revealed compatible interaction (Table 3). However, the lowest tested concentration of (+)-α-Pinene showed the highest biological index value (BI = 95). Overall, all the tested concentrations revealed strong compatible interaction by revealing BI > 79 (Table 3).

### 3.2. Date Palm Dust Mites Mortality Response against Pathogen-Toxin Interaction

The current study revealed that all the treatments whether a sole application or their combined application were lethal to the *O. afrasiaticus*. The increase in treatment concentration resulted in the enhanced mortality response among tested date palm dust mites (Table 4). The *B. bassiana* ARSEF 8465 conidia were deemed to be the least lethal treatment by revealing the highest LC_50_ value (LC_50_ = 19.16 mg/mL of the conidia of *B. bassiana* ARSEF 8465). However, mortality of *O. afrasiaticus* showed significant differences in response to conidial suspensions of *B. bassiana* ARSEF 8465 (Table 4) recorded at different time intervals (*F* = 64.79; *p* < 0.0001), concentrations (*F* = 771.36; *p* < 0.0001), and their interaction (*F* = 14.92; *p* < 0.0001). Similarly, sole application of (+)-α-Pinene also exhibited concentration-dependent mortality response (Table 4). The exposure of (+)-α-Pinene on the date palm leaf-disks at different concentrations (*F* = 1496.85; *p* < 0.0001), recorded mortality at different time intervals (*F* = 70.61; *p* < 0.0001), and their interaction (*F* = 82.94; *p* < 0.0001) revealed significant differences. The LC_50_ value was found to be 3.41 mg/mL.

The interaction of the conidial suspension of *B. bassiana* ARSEF 8465 with different concentrations of (+)-α-Pinene evaluated by four different Schemes revealed combined treatment enhanced mortality effect in all the studied interactions (Table 4). Only the Scheme I (20% (+)-α-Pinene: 80% *B. bassiana* ARSEF 8465 Conidia) bioassay results exhibited Antagonistic interaction by revealing a joint toxicity index score of 58 (Table 5). The mortality results of the compiled treatments for Scheme I showed the highest LC_50_ value (7.06 mg/mL), among all the interaction treatments (Table 5). However, mortality response at different time intervals (*F* = 144.35; *p* < 0.0001), with different concentrations (*F* = 611.98; *p* < 0.0001), and their interaction (*F* = 72.40; *p* < 0.0001), exhibited significant differences (Table 4).

There was a significant difference in the cumulative percent mortality (Table 4) of *O. afrasiaticus* following exposure to the Scheme II bioassay (LC_50_ = 4.20 mg/mL) different concentrations (*F* = 665.49; *p* < 0.0001), recorded at different time intervals (*F* = 221.62; *p* < 0.0001), and their interaction (*F* = 101.61; *p* < 0.0001). The Scheme II bioassay results revealed synergistic interaction (joint toxicity index = 121) as shown in Table 5.

The cumulative percent mortality of *O. afrasiaticus* treated with Scheme III bioassays treatments exhibited synergistic interaction by revealing high (280) joint toxicity index (Table 5). In addition, the combined treatment effect was recorded in terms of low LC_50_ value (2.40 mg/mL). However, different concentrations (*F* = 686.63; *p* < 0.0001), resulted in the significant differences in the mortality of *O. afrasiaticus* recorded at various time intervals (*F* = 432.56; *p* < 0.0001). Furthermore, interaction (*F* = 69.82; *p* < 0.0001), also revealed significant differences (Table 4).

The simultaneous exposure of (+)-α-Pinene and *B. bassiana* ARSEF 8465 at a proportion compiled in Scheme I increased the mortality of *O. afrasiaticus* (Table 4). The treatment effect in this Scheme I of bioassays were found to interact synergistically (joint toxicity index = 755). Furthermore, Probit analysis revealed the lowest LC_50_ value (1.32 mg/mL), compared with other interactions (Table 5). The analysis showed significant differences in the mortality of date palm dust mites recorded at different time intervals (*F =* 457.30; *p* < 0.0001), against different concentrations (*F* = 546.51; *p* < 0.0001), and their interaction (*F* = 66.19; *p* < 0.0001).

### 3.3. Defense-Related Enzymatic Activities Analysis

The catalase (CAT) activities of *O. afrasiaticus* measured 96 h post-exposure exhibited significant differences (*F* = 1394.69; *p* < 0.0001). Overall, the sole application of conidial suspension of *B. bassiana* ARSEF 8465 at different concentrations failed to induce very high CAT activities, and resulted in less than 10% CAT activities relative to control treatment (Table 6). However, different concentrations under each interaction scheme exhibited significant differences in the relative CAT activities (*F* = 333.01; *p* < 0.0001), and their interaction (*F* = 8.99; *p* < 0.0001).

The relative activities of Superoxide dismutase (SOD) measured among *O. afrasiaticus* exposed with various concentrations (*F* = 714.09; *p* < 0.0001), treatments, (*F* = 1294.71; *p* < 0.0001), and their interaction (*F* = 64.20; *p* < 0.0001) exhibited significant differences. The most effective treatment (80% (+)-α-Pinene: 20% Conidia) determined in the current study on the basis of lowest LC_50_ value (1.32 mg/mL) greatly induced the activities of SOD and kept at a higher level of significance in comparison with rest of the treatments (Table 6). On the contrary, relative CAT activities from *O. afrasiaticus* fed with sole application of (+)-α-Pinene, and the treatment with 20% (+)-α-Pinene: 80% Conidia, remained at a lower level of significance in comparison with the rest of the treatments.

Relative activities of glutathione S-transferase (GST) from *O. afrasiaticus* with various treatments (*F* = 311.25; *p* < 0.0001), compiled under different concentrations (*F* = 647.04; *p* < 0.0001), and their interaction (*F* = 27.28; *p* < 0.0001), exhibited highly significant differences (Table 6). The treatment with 20% (+)-α-Pinene: 80% conidia could not greatly induce GST activities, and therefore, the relative GST activities kept at lower level of significance in comparison with other treatments. Overall, combination 2.80 mg/mL (+)-α-Pinene + 4 mg/mL conidia greatly induced activities of GST, and kept at a high level of significance compared with all the treatments. Similarly, relative acetylcholinesterase (AChE) activities also produced a similar pattern by showing the highest AchE activities in response to 2.80 mg/mL (+)-α-Pinene + 4 mg/mL conidia treatment (Table 6). The relative AchE activities measured from date palm dust mites exposed with different treatments (*F* = 930.88; *p* < 0.0001), complied with different concentrations (*F* = 380.72; *p* < 0.0001), and their interaction (*F* = 11.88; *p* < 0.0001) showed highly significant differences (Table 6).

## 4. Discussion

The development of effective and stable pest management option involves the extensive toxin-pathogen compatibility and target host time- and concentration-dependent mortality response experimentation. This study clearly showed that the addition of (+)-α-Pinene found to be compatible with the *B. bassiana* ARSEF 8465, and their dual application found to be an attractive alternative approach to circumvent slow mortality criticism by producing strong synergistic interaction, which tremendously induced the target host defense-related enzymatic regulation, and ultimately increase the mortality of *O. afrasiaticus*.

Naturally-occurring entomopathogenic fungi are well-known to infect the populations of pests [43,45,46,47]. The infection of the conidial suspensions of *B. bassiana* ARSEF 8465 evaluated here against date palm dust mites exhibited the highest LC_50_ value (LC_50_ = 19.16 mg/mL). Furthermore, none of the tested conidial concentration able to impart 100% mortality, and their recorded cumulative mortality even at the highest concentration found to be <75%. The slow mortality response revealed here in the current study by the fungal conidial suspensions is in agreement with previous studies aiming to screen the most virulent isolate against various pest species [5,29,45]. These findings enabled us to suggest that slow fungal conidial mortality drawback could be overcome on one hand by reprogramming fungal virulence controlling metalloproteinases [48], or by incorporating compatible plant-based secondary compounds [6], for effective pest management.

Plant secondary metabolites are untapped chemicals reservoir currently emerged as an attractive management option for agricultural pests due to the advancement in analytical chemistry and least risk to the environment. The studied (+)-α-Pinene is a polyphenolic terpene hydrocarbon with known toxicity against various pests including head lice [31], *Sitophilus zeamais* Motschulsky [32], *Spodoptera litura* and *Achaea janata* [33]. The concentration-dependent time mortality response of *O. afrasiaticus* exhibited enhanced mortality response over time and concentrations in the current study. These results coincide with previous research findings [32] that strengthened our findings by revealing that α-Pinene is a promising compound for developing a novel effective formulation against *S. zeamais* Motschulsky. However, the low toxicity response (LC_50_ = 3.41 mg/mL) recorded in this study is in compliance with previous studies against date palm dust mites exposed with some other plant secondary metabolites including 1-chlorooctadecane [6], and phytol [37], and these findings suggested the necessity to develop compatible toxin-pathogen synergistic interaction. Although the (+)-α-Pinene differed significantly from the control, vegetative growth and percent conidial viability of *B. bassiana* ARSEF 8465 against all tested concentration was high, ranging from 95.00 to 98.20% (conidial germination), and 74.20 to 85.40 mm (vegetative growth). The biological index score (BI ranged from 79–95) of *B. bassiana* ARSEF 8465 calculated against each tested concentration of (+)-α-Pinene demonstrated that their joint application is suitable to develop synergistic interaction against *O. afrasiaticus* populations.

For successful *O. afrasiaticus* populations control in the date palm plantations, the importance of compatible toxin-pathogen interaction is highly applicable to facilitate the development of eco-friendly products. Our results revealed that pairing of *B. bassiana* ARSEF 8465 with (+)-α-Pinene in different proportions exhibited different responses among exposed date palm dust mites. However, the bioassay schemes with a higher proportion of (+)-α-Pinene contribute to enhanced mortality response, which ultimately lead to synergistic interaction. The enhanced mortality response among the treatments with a higher proportion of compounds recorded in the current study coincides with previous studies reporting the incorporation of phytol within the conidial suspensions tremendously enhance the combined treatment effect by revealing a very high score of joint toxicity index (691) [37]. The enhancement of treatment efficacy through the joint application of *B. bassiana* and commercial biopesticide against Colorado potato beetle larvae further strengthened the current findings [49], and allowed us to demonstrate that combined treatment combination added advantage by reducing mortality time and enhancing percent mortality. Similarly, the Scheme IV bioassays (80% (+)-α-Pinene: 20% *B. bassiana* ARSEF 8465 Conidia) depicted strong synergistic interaction (joint toxicity index = 755). These results are further supported by a previous study [50], which showed that the degree of synergy in terms of joint toxicity index increased, as the share of plant secondary metabolites increased in the joint application treatment comprising of conidial suspensions and plant secondary metabolites.

The joint application of conidial suspension and chemical compounds, which mainly aimed at improving the treatment effect might also lead to antagonistic interaction that limits the combination of the applied mixture [51]. In our study, the joint application under the Scheme I (20% (+)-α-Pinene: 80% *B. bassiana* ARSEF 8465 Conidia) showed enhanced mortality response compared with *O. afrasiaticus* exposed to (+)-α-Pinene or *B. bassiana* ARSEF 8465 sole application, but without synergistic interaction. However, both the tested fractions interacted antagonistically (joint toxicity index = 58) that might because of low proportion of (+)-α-Pinene. The current interaction data agree with Al-mazra’awi at al. [52], who showed that joint application of *B. bassiana* and neem tree extracts although showed high mortality, but revealed antagonistic interaction. Taken together, these findings suggested that careful synthesis of joint application is critically important to shift antagonistic interaction towards strong synergistic interaction to develop strong toxin pathogen synergy for effective and efficient management of agricultural pests.

Antioxidant defense system is important to scavenge the reactive oxygen species (ROS) generated within the target host as a result of the exposure to toxin/pathogen. The enzymes including SOD and CAT that prevent the accumulation of ROS explored in the current study depicted significantly high activities compared to control treatment after 96 h post-exposure. The increase in CAT and SOD activities is consistent with previous studies [37,43], revealing that the most potent treatment led to strong activities of antioxidant enzymes, which ultimately tremendously declined at the lateral stage of infection. These results are further strengthened by previous research investigations, revealing that increase in stress whether biotic or abiotic tremendously induced the activities of enzymes regulating antioxidant defense mechanism [43,53]. Furthermore, the degree of response of antioxidant enzymes modulated with the intensity of the stress to prevent ROS accumulation and maintain balance.

Host defense mechanisms in the context of toxin-pathogen interaction studies are very much pertinent because these interactions act as drivers to impart physiological stress among target hosts. Obtained results indicated that synergistic effect greatly induced the relative GST and AchE activities compared to control treatment. The enhanced defense-related enzymatic response revealed especially from the interaction treatments (synergistic interactions) with a higher proportion of % (+)-α-Pinene represents date palm dust mites response, which ultimately leads to strong physiological stress also confirmed by previous studies conducted on the host defense mechanism [54,55,56,57]. Furthermore, our results on the physiological impacts of (+)-α-Pinene and *B. bassiana* ARSEF 8465 conidia against date palm dust mites are consistent with those of Zibaee and Bandani [58], who reported significantly enhanced GST response among *Eurygaster integriceps* Puton exposed with *Artemisia annua*. These findings allowed us to suggest that the enzymes involved in defense mechanism play a pivotal role as these mechanisms utilize most of the host energy to defend the toxin or pathogen. The host could withstand these circumstances to certain limits and these conditions ultimately led the target host towards death.

## 5. Conclusions

In conclusion, the results of the current study demonstrated a compatible interaction between (+)-α-Pinene with *B. bassiana* ARSEF 8465 that greatly induced the activities of antioxidant (CAT and SOD), and defense-related (GST and AchE) enzymes, and ultimately imparted higher mortality of date palm dust mites. Therefore, the joint application of (+)-α-Pinene and *B. bassiana* ARSEF 8465 conidial suspensions based bio-control agents could provide a greater opportunity for environmentally friendly practical integrated pest management strategy for *Oligonychus afrasiaticus* (McGregor), an important economic pest of date palm fruit (dates).

## Figures and Tables

**Table 1:** Overview of *B. bassiana* ARSEF 8465 Biological Index Interactions.

Biological Index ^1^	Criterion	Classification
Conidia vs. (+)-α-Pinene	>66	Compatible
	42–66	Moderately Toxic
	<42	Toxic

^1^ BI was calculated using the formulae [47 × VG + 43 × SP + 10 × GR]/100.

**Table 2:** Overview of the (+)-α-Pinene and *B. bassiana* ARSEF 8465 combination bioassays to record the nature of their interaction.

(+)-α-Pinene (mg/mL).	Scheme I		Scheme II		Scheme III		Scheme IV		Conidia (mg/mL)
	Share of Category Weights		Share of Category Weights		Share of Category Weights		Share of Category Weights		
	20% (+)-α-Pinene: 80% Conidia		40% (+)-α-Pinene: 60% Conidia		60% (+)-α-Pinene: 40% Conidia		80% (+)-α-Pinene: 20% Conidia		
	(+)-α-Pinene (mg/mL)	Conidia mg/mL	(+)-α-Pinene (mg/mL)	Conidia mg/mL	(+)-α-Pinene (mg/mL)	Conidia mg/mL	(+)-α-Pinene (mg/mL)	Conidia mg/mL	
0.7	0.14	3.2	0.28	2.4	0.42	1.6	0.56	0.8	4
1.4	0.28	6.4	0.56	4.8	0.84	3.2	1.12	1.6	8
2.1	0.42	9.6	0.84	7.2	1.26	4.8	1.68	2.4	12
2.8	0.56	12.8	1.12	9.6	1.68	6.4	2.24	3.2	16
3.5	0.70	16.0	1.40	12.0	2.10	8.0	2.80	4.0	20

**Table 3:** Biological Index of *B. bassiana* ARSEF 8465 calculated from various growth parameters in response to different concentrations of (+)-α-Pinene.

Treatments (mg/mL)	Germination (%) *	Vegetative Growth (mm) *	Conidiation (×10^7^ Conidia/mL) *	Biological Index	Classification
Control	98.90 ± 0.50 ^a^	86.60 ± 1.98 ^a^	7.90 ± 0.66 ^a^	-	-
0.7	98.00 ± 0.96 ^ab^	85.40 ± 2.32 ^ab^	7.20 ± 0.59 ^ab^	95	Compatibility
1.4	97.60 ± 0.76 ^ab^	83.10 ± 1.69 ^ab^	6.70 ± 0.54 ^abc^	91	Compatibility
2.1	96.40 ± 0.80 ^bc^	80.40 ± 1.83 ^bc^	6.40 ± 0.56 ^abc^	88	Compatibility
2.8	95.90 ± 0.98 ^bc^	76.80 ± 2.06 ^cd^	5.80 ± 0.47 ^bc^	83	Compatibility
3.5	95.20 ± 1.19 ^c^	74.20 ±1.69 ^d^	5.30 ± 0.30 ^c^	79	Compatibility

* Means ± SE values with different letter(s) are significantly different (Fisher’s LSD test; α = 0.05).

**Table 4:** Cumulative percent mortality of date palm dust mites fed on date palm leaf-disk treated with various proportions of (+)-α-Pinene and *B. bassiana* ARSEF 8465 alone or in different combinations.

Treatments	Post-Exposure Duration		
	2 d	4 d	6 d
(+)-α-Pinene	(%)	(%)	(%)
0.7 mg/mL	08.40 ± 0.75 ^i^	12.40 ± 0.75 ^h^	24.40 ± 1.33 ^fg^
1.4 mg/mL	12.40 ± 0.98 ^h^	21.60 ± 1.33 ^g^	32.40 ± 2.14 ^e^
2.1 mg/mL	23.60 ± 1.72 ^g^	28.80 ± 2.06 ^ef^	49.20 ± 2.87 ^c^
2.8 mg/mL	27.60 ± 1.72 ^ef^	42.40 ± 1.83 ^d^	63.20 ± 2.33 ^b^
3.5 mg/mL	31.60 ± 1.94 ^e^	56.80 ± 2.15 ^b^	79.60 ± 2.48 ^a^
Scheme I: 20% (+)-α-Pinene: 80% Conidia			
0.14 mg/mL (+)-α-Pinene + 3.2 mg/mL conidia	19.60 ± 1.17 ^k^	36.40 ± 1.94 ^h^	66.40 ± 1.72 ^e^
0.28 mg/mL (+)-α-Pinene + 6.4 mg/mL conidia	24.40 ± 1.17 ^j^	43.20 ± 2.06 ^g^	72.40 ± 2.99 ^d^
0.42 mg/mL (+)-α-Pinene + 9.6 mg/mL conidia	28.00 ± 1.10 ^i^	54.40 ± 1.60 ^f^	77.60 ± 2.86 ^c^
0.56 mg/mL (+)-α-Pinene + 12.8 mg/mL conidia	29.20 ± 1.74 ^i^	65.60 ± 3.06 ^e^	83.60 ± 2.79 ^b^
0.70 mg/mL (+)-α-Pinene + 16.0 mg/mL conidia	32.80 ± 2.15 ^h^	76.40 ± 2.48 ^cd^	87.60 ± 2.71 ^a^
Scheme II: 40% (+)-α-Pinene: 60% Conidia			
0.28 mg/mL (+)-α-Pinene + 2.4 mg/mL conidia	21.60 ± 1.17 ^l^	41.60 ± 1.72 ^h^	70.40 ± 2.14 ^f^
0.56 mg/mL (+)-α-Pinene + 4.8 mg/mL conidia	23.60 ± 1.60 ^l^	43.20 ± 2.06 ^h^	80.80 ± 2.42 ^de^
0.84 mg/mL (+)-α-Pinene + 7.2 mg/mL conidia	28.40 ± 1.47 ^k^	61.20 ± 2.15 ^g^	84.40 ± 2.14 ^c^
1.12 mg/mL (+)-α-Pinene + 9.6 mg/mL conidia	31.60 ± 1.72 ^j^	77.60 ± 2.48 ^e^	89.60 ± 2.14 ^b^
1.40 mg/mL (+)-α-Pinene + 12.0 mg/mL conidia	34.80 ± 1.62 ^i^	81.60 ± 2.56 ^cd^	92.80 ± 1.85 ^a^
Scheme III: 60% (+)-α-Pinene: 40% Conidia			
0.42 mg/mL (+)-α-Pinene + 1.6 mg/mL conidia	23.60 ± 1.17 ^j^	46.60 ± 2.14 ^e^	77.20 ± 1.85 ^c^
0.84 mg/mL (+)-α-Pinene + 3.2 mg/mL conidia	27.60 ± 1.47 ^i^	71.20 ± 2.58 ^d^	87.60 ± 2.32 ^b^
1.26 mg/mL (+)-α-Pinene + 4.8 mg/mL conidia	34.40 ± 1.60 ^h^	87.20 ± 2.58 ^b^	96.40 ± 0.98 ^a^
1.68 mg/mL (+)-α-Pinene + 6.4 mg/mL conidia	38.40 ± 1.47 ^g^	97.60 ± 0.75 ^a^	98.80 ± 0.49 ^a^
2.10 mg/mL (+)-α-Pinene + 8.0 mg/mL conidia	40.40 ± 1.60 ^f^	98.80 ± 0.49 ^a^	99.20 ± 0.49 ^a^
Scheme IV: 80% (+)-α-Pinene: 20% Conidia			
0.56 mg/mL (+)-α-Pinene + 0.8 mg/mL conidia	24.40 ± 1.60 ^h^	51.60 ± 1.72 ^d^	80.80 ± 1.85 ^c^
1.12 mg/mL (+)-α-Pinene + 1.6 mg/mL conidia	29.60 ± 1.60 ^g^	88.00 ± 3.46 ^b^	96.80 ± 0.80 ^a^
1.68 mg/mL (+)-α-Pinene + 2.4 mg/mL conidia	36.00 ± 1.67 ^f^	94.80 ± 2.15 ^a^	97.20 ± 1.02 ^a^
2.24 mg/mL (+)-α-Pinene + 3.2 mg/mL conidia	42.80 ± 1.32 ^e^	98.80 ± 0.80 ^a^	98.80 ± 0.80 ^a^
2.80 mg/mL (+)-α-Pinene + 4.0 mg/mL conidia	43.20 ± 1.85 ^e^	99.20 ± 0.49 ^a^	99.60 ± 0.40 ^a^
*B. bassiana* ARSEF 8465 Conidia			
4 mg/mL	6.40 ± 0.75 ^j^	15.60 ± 1.17 ^hi^	24.40 ± 1.72 ^ef^
8 mg/mL	12.80 ± 1.02 ^i^	20.80 ± 1.02 ^fg^	31.60 ± 1.17 ^d^
12 mg/mL	19.20 ± 1.02 ^gh^	32.40 ± 3.97 ^d^	42.40 ± 1.72 ^c^
16 mg/mL	22.40 ± 1.47 ^f^	44.40 ± 1.72 ^c^	52.80 ± 2.33 ^b^
20 mg/mL	26.40 ± 1.33 ^de^	56.40 ± 2.79 ^b^	69.60 ± 2.79 ^a^

Means ± SE values with different letter(s) within each scheme at various time intervals are significantly different (Fisher’s LSD test; α = 0.05).

**Table 5:** Interaction (Synergistic or Antagonistic) between (+)-α-Pinene and *B. bassiana* ARSEF 8465.

Schemes	* LC_50_ (mg/mL)	Joint Toxicity	Interaction **
Scheme IV: 80% (+)-α-Pinene: 20% *B. bassiana* ARSEF 8465 Conidia			
0.56 mg/mL (+)-α-Pinene + 0.8 mg/mL conidia	1.32 (1.09 to 1.51)	755	Synergistic
1.12 mg/mL (+)-α-Pinene + 1.6 mg/mL conidia			
1.68 mg/mL (+)-α-Pinene + 2.4 mg/mL conidia			
2.24 mg/mL (+)-α-Pinene + 3.2 mg/mL conidia			
2.80 mg/mL (+)-α-Pinene + 4.0 mg/mL conidia			
Scheme III: 60% (+)-α-Pinene: 40% *B. bassiana* ARSEF 8465 Conidia			
0.42 mg/mL (+)-α-Pinene + 1.6 mg/mL conidia	2.40 (2.02 to 2.74)	280	Synergistic
0.84 mg/mL (+)-α-Pinene + 3.2 mg/mL conidia			
1.26 mg/mL (+)-α-Pinene + 4.8 mg/mL conidia			
1.68 mg/mL (+)-α-Pinene + 6.4 mg/mL conidia			
2.10 mg/mL (+)-α-Pinene + 8.0 mg/mL conidia			
Scheme II: 40% (+)-α-Pinene: 60% *B. bassiana* ARSEF 8465 Conidia			
0.28 mg/mL (+)-α-Pinene + 2.4 mg/mL conidia	4.20 (3.16 to 5.07)	121	Synergistic
0.56 mg/mL (+)-α-Pinene + 4.8 mg/mL conidia			
0.84 mg/mL (+)-α-Pinene + 7.2 mg/mL conidia			
1.12 mg/mL (+)-α-Pinene + 9.6 mg/mL conidia			
1.40 mg/mL (+)-α-Pinene + 12.0 mg/mL conidia			
Scheme I: 20% (+)-α-Pinene: 80% *B. bassiana* ARSEF 8465 Conidia			
0.14 mg/mL (+)-α-Pinene + 3.2 mg/mL conidia	7.06 (5.63 to 8.44)	58	Antagonistic
0.28 mg/mL (+)-α-Pinene + 6.4 mg/mL conidia			
0.42 mg/mL (+)-α-Pinene + 9.6 mg/mL conidia			
0.56 mg/mL (+)-α-Pinene + 12.8 mg/mL conidia			
0.70 mg/mL (+)-α-Pinene + 16.0 mg/mL conidia			

* LC_50_ stands for lethal concentration to kill 50% of the population; ** joint toxicity ≥100 is classified as synergistic interaction, while, Joint toxicity < 100 is classified as antagonistic interaction.

**Table 6:** Relative enzymatic activities of *O. afrasiaticus* fed on leaves treated with various proportions of (+)-α-Pinene and *B. bassiana* ARSEF 8465 alone or in different combinations for comparative host defense-related regulation analysis.

Treatments	Relative Enzymatic Activities (%)			
	CAT	SOD	GST	AchE
*B. bassiana* ARSEF 8465 Conidia				
4 mg/mL	2.67 ± 0.23 ^n^	9.67 ± 0.25 ^opq^	7.69 ± 0.74 ^no^	1.18 ± 0.29 ^m^
8 mg/mL	4.45 ± 0.28 ^n^	11.53 ± 0.21 ^no^	11.66 ± 0.93 ^klm^	2.62 ± 0.37 ^jklm^
12 mg/mL	8.33 ± 0.98 ^m^	15.20 ± 0.20 ^lm^	13.49 ± 1.24 ^ijk^	3.92 ± 0.48 ^hij^
16 mg/mL	9.06 ± 0.71 ^m^	17.87 ± 1.42 ^k^	15.38 ± 1.97 ^hij^	5.44 ± 0.65 ^fgh^
20 mg/mL	10.44 ± 0.69 ^m^	19.13 ± 0.60 ^k^	21.87 ± 0.34 ^g^	12.98 ± 1.52 ^d^
(+)-α-Pinene				
0.7 mg/mL	19.55 ± 0.83 ^k^	1.24 ± 0.13 ^u^	9.04 ± 0.45 ^mn^	2.14 ± 0.35 ^klm^
1.4 mg/mL	23.27 ± 0.44 ^j^	1.79 ± 0.15 ^tu^	16.02 ± 1.59 ^hi^	3.58 ± 0.30 ^ijk^
2.1 mg/mL	28.07 ± 0.62 ^i^	7.09 ± 1.22 ^qr^	21.87 ± 0.34 ^g^	5.92 ± 0.78 ^fg^
2.8 mg/mL	35.71 ± 1.04 ^h^	9.67 ± 0.25 ^opq^	26.78 ± 0.32 ^f^	9.11 ± 0.48 ^e^
3.5 mg/mL	36.46 ± 1.04 ^h^	14.17 ± 1.62 ^mn^	39.24 ± 1.20 ^d^	14.62 ± 1.08 ^d^
Scheme I: 20% (+)-α-Pinene: 80% Conidia				
0.14 mg/mL (+)-α-Pinene + 3.2 mg/mL conidia	10.85 ± 1.99 ^m^	3.33 ± 0.55 ^stu^	4.59 ± 0.88 ^o^	1.59 ± 0.36 ^lm^
0.28 mg/mL (+)-α-Pinene + 6.4 mg/mL conidia	14.80 ± 1.08 ^l^	4.37 ± 0.96 ^st^	7.40 ± 0.96 ^no^	2.18 ± 0.27 ^jklm^
0.42 mg/mL (+)-α-Pinene + 9.6 mg/mL conidia	23.81 ± 0.91 ^j^	5.45 ± 0.57 ^rs^	10.08 ± 0.85 ^lmn^	3.56 ± 0.47 ^ijk^
0.56 mg/mL (+)-α-Pinene + 12.8 mg/mL conidia	27.79 ± 0.73 ^i^	7.64 ± 1.19 ^pqr^	13.24 ± 0.87 ^ijk^	4.86 ± 0.59 ^ghi^
0.70 mg/mL (+)-α-Pinene + 16.0 mg/mL conidia	29.12 ± 0.50 ^i^	9.32 ± 0.31 ^opq^	17.59 ± 1.01 ^h^	6.03 ± 0.31 ^fg^
Scheme II: 40% (+)-α-Pinene: 60% Conidia				
0.28 mg/mL (+)-α-Pinene + 2.4 mg/mL conidia	43.08 ± 0.82 ^g^	9.79 ± 0.26 ^op^	13.05 ± 0.98 ^ijkl^	3.16 ± 0.63 ^ijkl^
0.56 mg/mL (+)-α-Pinene + 4.8 mg/mL conidia	44.12 ± 0.96 ^g^	20.10 ± 0.24 ^k^	15.59 ± 1.28 ^hij^	7.13 ± 0.46 ^f^
0.84 mg/mL (+)-α-Pinene + 7.2 mg/mL conidia	49.70 ± 0.63 ^f^	24.09 ± 0.50 ^j^	24.73 ± 1.12 ^fg^	9.04 ± 0.53 ^e^
1.12 mg/mL (+)-α-Pinene + 9.6 mg/mL conidia	58.19 ± 0.81 ^d^	27.30 ± 0.69 ^i^	35.61 ± 2.84 ^e^	13.41 ± 0.83 ^d^
1.40 mg/mL (+)-α-Pinene + 12.0 mg/mL conidia	63.86 ± 1.89 ^c^	39.06 ± 0.56 ^g^	44.30 ± 2.42 ^c^	19.61 ± 1.10 ^c^
Scheme III: 60% (+)-α-Pinene: 40% Conidia				
0.42 mg/mL (+)-α-Pinene + 1.6 mg/mL conidia	44.12 ± 0.96 ^g^	17.44 ± 0.32 ^kl^	12.67 ± 0.81 ^jkl^	6.49 ± 0.36 ^fg^
0.84 mg/mL (+)-α-Pinene + 3.2 mg/mL conidia	49.89 ± 1.62 ^f^	26.54 ± 0.72 ^ij^	18.31 ± 0.86 ^h^	7.15 ± 0.44 ^f^
1.26 mg/mL (+)-α-Pinene + 4.8 mg/mL conidia	57.30 ± 1.11 ^d^	35.71 ± 1.04 ^h^	27.93 ± 1.40 ^f^	9.90 ± 0.70 ^e^
1.68 mg/mL (+)-α-Pinene + 6.4 mg/mL conidia	68.07 ± 0.98 ^b^	49.70 ± 0.63 ^e^	39.68 ± 1.47 ^d^	14.68 ± 0.76 ^d^
2.10 mg/mL (+)-α-Pinene + 8.0 mg/mL conidia	71.84 ± 2.05 ^a^	57.47 ± 1.02 ^c^	53.18 ± 1.46 ^b^	22.85 ± 0.88 ^b^
Scheme IV: 80% (+)-α-Pinene: 20% Conidia				
0.56 mg/mL (+)-α-Pinene + 0.8 mg/mL conidia	53.89 ± 1.11 ^e^	25.73 ± 1.83 ^ij^	17.59 ± 1.01 ^h^	9.71 ± 0.54 ^e^
1.12 mg/mL (+)-α-Pinene + 1.6 mg/mL conidia	57.13 ± 1.21 ^d^	43.08 ± 0.82 ^f^	25.29 ± 1.53 ^f^	13.11 ± 0.74 ^d^
1.68 mg/mL (+)-α-Pinene + 2.4 mg/mL conidia	66.27 ± 1.19 ^bc^	52.42 ± 0.57 ^d^	40.33 ± 1.30 ^d^	19.89 ± 0.99 ^c^
2.24 mg/mL (+)-α-Pinene + 3.2 mg/mL conidia	66.61 ± 1.20 ^bc^	63.89 ± 1.88 ^b^	53.91 ± 1.29 ^b^	22.20 ± 0.64 ^b^
2.80 mg/mL (+)-α-Pinene + 4.0 mg/mL conidia	72.36 ± 1.89 ^a^	75.22 ± 2.02 ^a^	61.09 ± 0.89 ^a^	30.07 ± 1.09 ^a^

Means ± SE followed by different letter(s) within each column are significantly different (Fisher’s LSD test, α *=* 0.05).
